# Approaches to Study Native Chromatin-Modifying Complex Activities and Functions

**DOI:** 10.3389/fcell.2021.729338

**Published:** 2021-09-16

**Authors:** Maxime Galloy, Catherine Lachance, Xue Cheng, Félix Distéfano-Gagné, Jacques Côté, Amelie Fradet-Turcotte

**Affiliations:** ^1^St-Patrick Research Group in Basic Oncology, Oncology Division, Centre Hospitalier Universitaire (CHU) de Québec-Université Laval Research Center, Québec, QC, Canada; ^2^Department of Molecular Biology, Medical Biochemistry and Pathology, Laval University Cancer Research Center, Université Laval, Québec, QC, Canada

**Keywords:** chromatin, histone modification, native protein complexes, recombinant nucleosomes, nuclear protein depletion

## Abstract

The modification of histones—the structural components of chromatin—is a central topic in research efforts to understand the mechanisms regulating genome expression and stability. These modifications frequently occur through associations with multisubunit complexes, which contain active enzymes and additional components that orient their specificity and read the histone modifications that comprise epigenetic signatures. To understand the functions of these modifications it is critical to study the enzymes and substrates involved in their native contexts. Here, we describe experimental approaches to purify native chromatin modifiers complexes from mammalian cells and to produce recombinant nucleosomes that are used as substrates to determine the activity of the complex. In addition, we present a novel approach, similar to the yeast anchor-away system, to study the functions of essential chromatin modifiers by quickly inducing their depletion from the nucleus. The step-by-step protocols included will help standardize these approaches in the research community, enabling convincing conclusions about the specificities and functions of these crucial regulators of the eukaryotic genome.

## Introduction

Understanding chromatin structure and function has been a focus of intense research for decades. It is now well established that the chromatin plays primary active roles regulating genome-related processes, including gene-specific expression, DNA damage repair, and DNA replication during cell division (Allis and Jenuwein, 2016). Every few years, breakthrough discoveries propel the field into new territories, expanding our understanding of the molecular mechanisms involved and providing crucial insights on human diseases like cancer, an illness with combined genetic and epigenetic origins (Shen and Laird, 2013). This has led to an impressive surge in the development of epigenetic approaches to treat cancer (Brien et al., 2016). The research breakthroughs that have allowed these leaps have been conceptual, such as the identification of histone writers/readers/erasers, as well as technological, including approaches to study genome organization and modifications with increasing precision.

The epigenetic language written on the chromatin has been studied for over 20 years, and new mechanistic understandings continuously emerge. The post-translational modifications (PTMs) of conserved histone residues are diverse, and their combinations form specific signatures that can be read by effectors. As the same PTM can have opposing functions when added on different residues/histones, it is crucial to clearly identify and characterize the chromatin modifiers regulating each specific modified histone residue, as well as the reader proteins that recognize them. Historically, many studies have used histone peptides or free histones to identify the enzymes responsible for specific PTMs, as well as recombinant proteins instead of enzymes in their native forms. However, the specificities identified in these studies are often significantly different from those observed *in vivo* or when assayed *in vitro* on chromatin substrates (reviewed in Lalonde et al., 2014). In fact, true native specificity can only be reproduced in a test tube using native substrates, nucleosomal histones/chromatin, and enzymes in their physiological contexts, including any associated factors. It is clearly established that the histone residue specificity of chromatin modifiers can be determined by associated factors within large enzyme-containing protein complexes (Lalonde et al., 2014). In addition, associated reader modules within these large complexes further regulate the specificity of the modifiers by mediating crosstalk between different histone modifications (Lalonde et al., 2014).

In parallel, determining chromatin modifier specificity *in vivo* can be difficult because of their possible indirect effects on the modifications of other specific residues. Indeed, many histone PTMs are regulated by crosstalk between histone modifications, a phenomenon that can induce secondary effects when histone modifiers are depleted or knocked out (McGinty et al., 2008; Lalonde et al., 2014; Jacquet et al., 2016; Wojcik et al., 2018; Zhang and Kutateladze, 2019). This is a major reason why both *in vivo* and *in vitro* experiments are required to truly understand the intricate molecular mechanisms regulating chromatin modifiers.

## Approaches to Characterize Chromatin-Modifying Enzyme Activities and Functions

Over the years, multiple approaches to purify native protein complexes from mammalian nuclear extracts (NEs) have been developed. Several chromatin remodeling and modifying complexes have been efficiently purified to near homogeneity by introducing transgenes encoding tagged components. Tandem affinity purification (TAP) was developed more than 20 years ago in yeast and quickly transferred to higher eukaryotes (Rigaut et al., 1999; Puig et al., 2001). Different tag combinations have been tested, some of which provide high specificity and efficient elutions/high yields in native conditions. For example, the FLAG epitope has remained front and center for many years (Hopp et al., 1988; Einhauer and Jungbauer, 2001). However, one of the main issues with this approach is the formation of artefactual associations due to overexpression of the transgene compared to the physiological protein level (Gibson et al., 2013; Lalonde et al., 2014). This often occurs when proteins with significant homology (paralogs) to specific subunits or even the tagged protein are expressed. Keeping expression at a near-physiological level is possible, by aiming for single-copy genome integration and using different promoter strengths [e.g., using retroviral vectors at low multiplicity of infection or the Flp-In^TM^ system (Thermo Fisher Scientific)]. Random genome integration can also create problems due to well-established positional effects on gene expression depending on the local chromatin state. Thus, achieving near-physiological expression can require isolating and characterizing multiple clones. The development of safe harbors for transgene integration has great advantages because the different cell lines created are isogenic, as in lower eukaryotes. The *AAVS1* and *ROSA26* loci have been frequently used for this purpose, and recent genome editing tools have made this endeavor quite straightforward (DeKelver et al., 2010; Sadelain et al., 2012; Dalvai et al., 2015). Of course, the gold standard of reproducing physiological conditions is to tag the endogenous gene and use it to purify the native chromatin modifying complex. This can now also be achieved, thanks to the power of clustered regularly interspaced short palindromic repeats (CRISPR)/Cas9-based genome editing (Dalvai et al., 2015).

To obtain native chromatin substrates, several protocols are available to purify endogenous chromatin from human cell lines (Côté et al., 1995; Utley et al., 1996; Schnitzler, 2000). Substrates are produced by micrococcal nuclease digestion and can be fractionated in different lengths, from long oligonucleosomes to mononucleosomes. They have several advantages: they already contain the vast majority of known histone marks, reproduce the natural substrate, and enable binding specificity analysis. However, these marks are present at low stoichiometries on each residue, and the DNA sequences associated with the nucleosomes are heterogeneous. Using recombinant histones to assemble nucleosome core particles (NCPs) is a powerful alternative to producing chromatin substrates with defined DNA and histone status. The “Widom” sequence is extremely efficient for nucleosome assembly/positioning using recombinant histone octamers (Lowary and Widom, 1998; Thåström et al., 1999). The resulting recombinant nucleosomes are homogeneous, and their main advantage is that they can be enzymatically or chemically modified to introduce a specific histone PTM at a given residue with high stoichiometry. This allows very clear analysis of crosstalk occurring between different histone marks, via specific reader proteins, or within chromatin remodeling/modifying complexes. Over the past few years, the use of purified native chromatin modifying complexes and recombinant nucleosomes has produced exquisite high-resolution 3D structures of chromatin-bound complexes, which have provided crucial mechanistic insights (Poepsel et al., 2018; Patel et al., 2019; Farnung et al., 2020; Wagner et al., 2020; Wang et al., 2020).

*In vivo* loss of function analysis of chromatin modifiers has relied mostly on RNA interference-mediated depletion and mouse gene knockouts. However, these can create indirect and/or downstream effects that mask the primary role of the enzyme being studied (discussed in Lalonde et al., 2014). For example, depletion of chromatin modifiers such as the HBOI acetyltransferase, impact cell cycle progression into S phase (Doyon et al., 2006; Miotto and Struhl, 2010; Havasi et al., 2013; Feng et al., 2016). As many chromatin modifiers are essential for cell viability or normal growth and because several histone marks are regulated during the cell cycle (Ma et al., 2015), changes due to of knockdown of such factors can mislead the investigator into linking a chromatin modification to a specific enzyme (Lalonde et al., 2014). The development of rapid depletion approaches, as used in lower eukaryotes, can bypass these problems. The auxin-inducible degron has proved popular; however, the tag is known to affect protein stability even in the absence of auxin, which can be alleviated by expressing transport inhibitor response 1 (Nishimura et al., 2020; Yesbolatova et al., 2020). The recently described degradation TAG does not seem to have the same problem, and efficiently and suddenly targets proteins to the proteasome (Nabet et al., 2018).

In this method article, we present up-to-date detailed protocols related to the approaches discussed above. First, we describe the use of the 3 × FLAG-2 × Strep tag fused to a gene of interest (either expressed from *AAVS1* or endogenously tagged) to purify native chromatin modifying complexes for biochemical/enzymatic assays. Second, we provide a step-by-step protocol for the production of recombinant mono- and di-nucleosomes, the latter having particular potential since recent studies have highlighted the importance of linker DNA in the mechanisms of many remodelers and modifiers (Poepsel et al., 2018; Bhardwaj et al., 2020). Finally, we present a new rapid depletion approach for mammalian cells, inspired by the yeast anchor-away system (Haruki et al., 2008). In this system, a nuclear protein is tagged with a peptide that can be induced to bind a tagged endogenous ribosomal protein, which acts as an anchor to rapidly export it to the cytosol, crippling its nuclear function.

## Step-By-Step Procedures

### Purification of Endogenously Tagged NuA4/TIP60 Complex (Figure 1)

**FIGURE 1:**
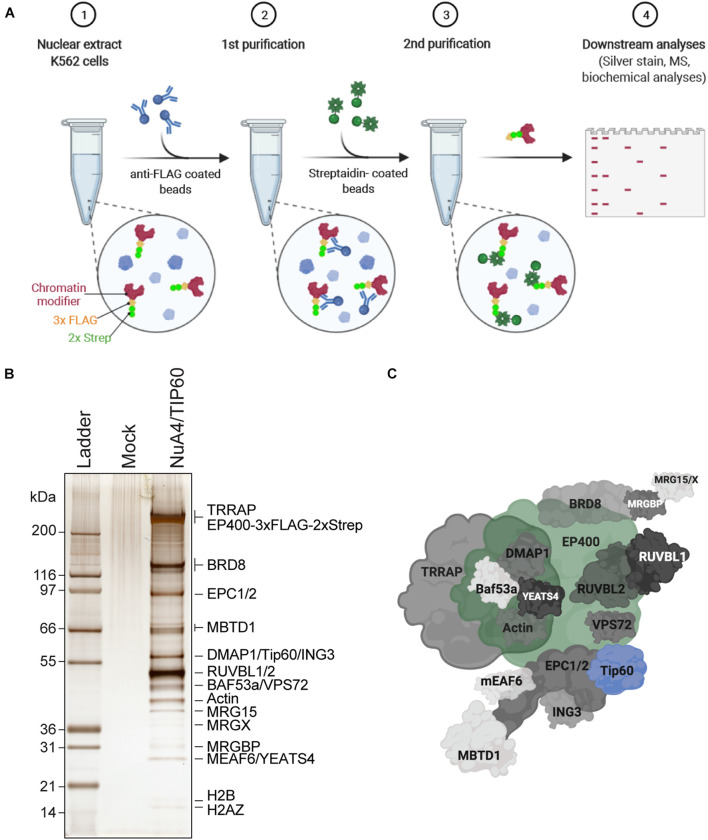
Purification of endogenously tagged native chromatin modifying enzymes **(A)** TAP. Step 1: Nuclear extracts are isolated from engineered K562 cell lines (here, C-terminally 3 × FLAG-2 × Strep-tagged endogenous EP400 was used as an example). Step 2: FLAG-Strep-tagged proteins are immunoprecipitated using anti-FLAG-coated beads and eluted with 3 × FLAG peptides. Step 3: Eluted FLAG-Strep-tagged proteins are isolated using streptavidin-coated beads and eluted with biotin. Step 4: Purified FLAG-Strep-tagged proteins are either analyzed by silver staining and/or MS, or directly used in biochemical assays. Created with BioRender.com. **(B)** Silver-stained purified NuA4/TIP60 native complex after TAP of 3 × FLAG-2 × Strep-EP400. K562 cells expressing the 3 × FLAG-2 × Strep tag alone (mock) were used as a control purification. **(C)** Schematic representation of the NuA4/TIP60 complex. The TIP60 subunit, which encompasses the acetyltransferase activity of the complex, is colored in blue and the tagged subunit EP400 is colored in green. Created with BioRender.com.

Tagging an endogenous gene to purify its encoded protein has the advantage of reflecting its physiological expression and regulation. Insertion of the small 3 × FLAG-2 × Strep tag using CRISPR/Cas9 and donor DNA is efficient, does not require a selection marker, and creates very limited sequence perturbation of the 5′ or 3′ untranslated regions. NuA4/TIP60 complex is a highly conserved multisubunit complex essential for cell cycle progression, gene-specific regulation, and DNA repair (Doyon et al., 2004). Here, the E1A binding protein p400 (EP400) NuA4/TIP60 was selected for endogenous tagging as it was successfully reported to be efficient to purify the native complex (Dalvai et al., 2015). However, the tagging of many other subunits of this complex has been successfully used to purified native complex (Ikura et al., 2000; Doyon et al., 2004, 2006; Dalvai et al., 2015; Jacquet et al., 2016). The generation of endogenously tagged EP400 in K562 cells was performed essentially as described in the third protocol presented here, and was reported in Dalvai et al. (2015). In this section, we describe the protocol for TAP of 3 × FLAG-2 × Strep-tagged native complexes from nuclear extracts (NEs) (Figure 1A).

1. ***NE preparation from K562 cells expressing endogenous 3×FLAG-2×Strep-EP400***

A. Large-scale expansion of K562 cells

i. Grow 1–3 L of K562 cells in spinner flasks with gentle agitation in basal RPMI medium supplemented with 25 mM HEPES-NaOH pH 7.4.
ii. Monitor cell growth and viability daily, carefully maintaining cultures between 2 × 10^5^ and 8 × 10^5^ cells/mL.
iii. Harvest cells at or slightly below 8 × 10^5^ cells/mL, before they reach confluency.
iv. Pellet the cells in a preparative centrifuge (700 × *g*, 10 min, 4°C). Resuspend and pool the pellets in 50 mL of cold phosphate-buffered saline (PBS). Centrifuge (700 × *g*, 10 min, 4°C), place the pellet on ice, and immediately prepare the NE.

B. NE preparation (as previously described in Abmayr et al., 2006)

i. Wash the cell pellets by adding four packed cell volumes of hypotonic buffer (10 mM HEPES pH 7.9, 1.5 mM MgCl_2_, and 10 mM KCl, with 0.2 mM phenylmethylsulfonyl fluoride (PMSF) and 0.5 M dithiothreitol (DTT) added just before use). Centrifuge (1,900 × *g*, 5 min, 4°C), remove the supernatant quickly, and resuspend well with three packed cell volumes of hypotonic buffer. Incubate on ice 10 min.
ii. Transfer the cells to a glass Dounce homogenizer with a type B pestle. Homogenize by douncing 15 times, then centrifuge (3,500 × *g*, 10 min, 4°C).
iii. Collect the supernatant (i.e., S-100 cytoplasmic extract) and estimate the packed nuclear volume of the pellet using the gradations on the conical tube.
iv. Add half the packed nuclear volume of low salt buffer (10 mM HEPES pH 7.9, 1.5 mM MgCl_2_, 20 mM KCl, 25% glycerol, 0.2 mM ethylenediaminetetraacetic acid (EDTA), with 0.2 mM PMSF and 0.5 M DTT added fresh) and resuspend well with gentle vortexing. Then, extract the soluble proteins by adding half the packed nuclear volume of high salt buffer (10 mM HEPES pH 7.9, 1.5 mM MgCl_2_, 1.2 M KCl, 25% glycerol, and 0.2 mM EDTA, with 0.2 mM PMSF and 0.5 M DTT added fresh) dropwise with gentle vortexing.
v. Dounce twice using a Dounce homogenizer with a type B pestle and incubate on a nutator for 30 min at 4°C.
vi. Pellet the extracted nuclei by ultracentrifugation (100,000 × *g*, 1 h, 4°C). Quickly transfer the supernatant (i.e., the NE) to a new Falcon tube.
vii. Snap freeze the NE in liquid nitrogen and store at −80°C.

2. ***TAP of EP400*** (as described in Doyon and Côté, 2016)
i. Thaw the NE^∗^ on ice, adjust to 0.1% Tween-20 (using a 10% stock), and centrifuge (40,000 × *g*, 1 h, 4°C).
ii. Preclear the NE using 250 μL Sepharose CL-6B resin prewashed with PBS and equilibrated with TAP buffer (20 mM HEPES-KOH pH 7.9, 300 mM KCl, 1.5 mM MgCl_2_, 0.2 mM EDTA, and 10% glycerol, with 10 mM sodium butyrate, 10 mM β-glycerophosphate, 1 mM PMSF, 5 mM NaF, 100 μM orthovanadate, 2 μg/mL leupeptin, 2 μg/mL pepstatin, and 5 μg/mL aprotinin added fresh) in a 10 mL Poly-Prep chromatography column. Collect the precleared NE in a 15 mL tube.
iii. Add 250 μL anti-FLAG M2 affinity beads to the precleared NE and incubate for 2 h at 4°C with rotation.
iv. Transfer to a 10 mL Poly-Prep chromatography column, harvest the flowthrough (FLAG-FT), and pass it through the column again. Wash the beads with 40 column volumes (CVs) of TAP buffer, then 40 CVs of TAP wash buffer #1 (20 mM HEPES-KOH pH 7.9, 300 mM KCl, 0.1% Tween-20, and 10% glycerol, with 1 mM DTT, 10 mM sodium butyrate, 10 mM β-glycerophosphate, 1 mM PMSF, 5 mM NaF, 100 μM orthovanadate, 2 μg/mL leupeptin, 2 μg/mL pepstatin, and 5 μg/mL aprotinin added fresh), followed by 40 CVs of TAP wash buffer #2 (20 mM HEPES-KOH pH 7.9, 150 mM KCl, 0.1% Tween-20, and 10% glycerol, with 1 mM DTT, 10 mM sodium butyrate, 10 mM β-glycerophosphate, 1 mM PMSF, 5 mM NaF, 100 μM orthovanadate, 2 μg/mL leupeptin, 2 μg/mL pepstatin, and 5 μg/mL aprotinin added fresh).
v. Transfer the beads in a 1.5 mL Eppendorf tube. Use TAP wash buffer #2 to rinse the column and collect all the beads. Centrifuge (239 × *g*, 5 min, 4°C) and carefully remove the supernatant.
vi. Elute the complex with 2.5 CVs of TAP wash buffer #2 supplemented with 200 μg/mL 3×FLAG peptide for 1 h at 4°C on a rotator.
vii. Centrifuge (250 × *g*, 5 min, 4°C) and carefully transfer the supernatant into a Micro Bio-Spin column placed in a 2 mL microcentrifuge tube. Centrifuge (250 × g, 1 min, 4°C) to collect the eluate. Collect a 15 μL sample to resolve by sodium dodecyl sulfate (SDS)-polyacrylamide gel electrophoresis (PAGE; FLAG first elution).
viii. Repeat steps vi and vii.
ix. Pool the FLAG elutions and add 125 μL Strep-Tactin Superflow Sepharose affinity matrix prewashed with 1 mL PBS followed by 1 mL TAP wash buffer #2. Incubate for 1 h at 4°C on a rotator.
x. Centrifuge (250 × *g*, 5 min, 4°C) and remove the flowthrough.
xi. Wash the beads three times with 1 mL TAP wash buffer #2.
xii. Elute the complex with 1 CV of TAP wash buffer #2 supplemented with 5 mM D-biotin for 1 h at 4°C on a rotator.
xiii. Centrifuge (250 × *g*, 5 min, 4°C) and carefully transfer the supernatant into a Micro Bio-Spin column placed in a 2 mL microcentrifuge tube. Centrifuge (250 × *g*, 1 min, 4°C) to collect the eluate. Aliquot a 15 μL sample for SDS-PAGE (biotin elution).
xiv. Repeat steps xiii and xiv.
xv. Aliquot the purified complex. Snap freeze in liquid nitrogen and keep at −80°C.

^∗^*NEs should always be kept on ice, and all purification steps should be performed at 4*°*C in a cold room.*

3. ***Analysis of EP400 complex subunits by SDS-PAGE and silver staining (Figures 1B,C)***
i. Load 15 μL of the FLAG and biotin elution fractions on a NuPage/Bolt 4–12% Bis-Tris gel and run for 42 min at 200 V in MOPS SDS running buffer (50 mM MOPS, 50 mM Tris, 0.1% SDS, and 1 mM EDTA).
ii. Incubate the gel for 1 h in 50% methanol, then for 30 min in 10% MeOH/7% acetic acid, and finally for 30 min in 10% glutaraldehyde.
iii. Wash the gel at least four times for 30 min with ultrapure water and let it soak in water overnight.
iv. The next day, change the ultrapure water 2–3 times before staining.
v. Incubate the gel with 5 μg/mL DTT for 30 min.
vi. Stain the gel with 0.1% (w/v) silver nitrate in ultrapure water for 30 min.
vii. Rinse twice with ultrapure water.
viii. Condition the gel with two rapid washes in carbonate developing solution (283 mM sodium carbonate, 0.0185% formaldehyde), then incubate in exactly 160 mL of solution.
ix. Stop the reaction when proper staining is attained by adding 8 mL of 2.3 M citric acid.
4. ***Sample preparation for mass spectrometry (MS) analysis***
i. Load 50 μL samples of the biotin elutions onto a Bolt 12% Bis-Tris gel and let run for >1 cm (approximately 4 min) at 200V to retain all the proteins in one band.
ii. Wash the gel briefly with ultrapure water and incubate it for 30 min in 10% MeOH/7% acetic acid.
iii. Incubate the gel O/N in Sypro Ruby gel stain.
iv. Rinse twice with ultrapure water and cut out the protein bands under UV light.
v. Rinse the bands twice with 70% acetonitrile and store the samples at −80°C.

## Characterization of *in vitro* Histone Acetyltranferase (HAT) Activity (Figure 2)

Chromatin modifiers like NuA4/TIP60 efficiently acetylate histone tails as well as purified histones; however, the use of oligonucleosomes purified from cell nuclei (Côté et al., 1995; Utley et al., 1996) or reconstituted from recombinant histones revealed different specificities toward these substrates (Lalonde et al., 2014). In this section, we describe a robust method used to reconstitute mono- and dinucleosomes from recombinant histones purified from *Escherichia coli*. The method is adapted (Dyer et al., 2003) and can be used to assemble recombinant nucleosome core particles (rNCPs) containing different types of histones. Here, rNCPs were reconstituted using untagged human H2A/H2B and *Xenopus laevis* H3/H4. Cysteine 110 of histone H3 was replaced with an alanine to block undesired cysteine alkylation in assays where analogs are used to label other residues (e.g., H4 K20Cme) (Simon, 2010). Histones tagged on their N-termini with tags such as histidine (His) or FLAG can also be used to assemble rNCPs; however, we have noted that on H2A, these tags interfere with NuA4/TIP60 activity *in vitro (data not shown)*. These observations are in line with previous studies showing that NuA4 binds the N-terminal tail of histone H2A (Huang and Tan, 2013). Here, two DNA fragments were used for reconstitution: a 151 bp fragment that contains a single copy of the 601 DNA used to assemble mononucleosomes (Lowary and Widom, 1998; Thåström et al., 1999; Dyer et al., 2003) and a 388 bp fragment containing two copies of 601 separated by a 48-bp linker DNA to assemble dinucleosomes (Kato et al., 2017; Machida et al., 2018). Note that an array of DNA fragments can be used to reconstitute rNCPs, facilitating structural studies of nucleosome assembly (Engeholm et al., 2009; Muthurajan et al., 2016). In this section, we also describe the main steps required to assess the activity of the purified NuA4/TIP60 complex *in vitro* using gel- and liquid-based assays (Côté et al., 1995; Utley et al., 1996).

**FIGURE 2:**
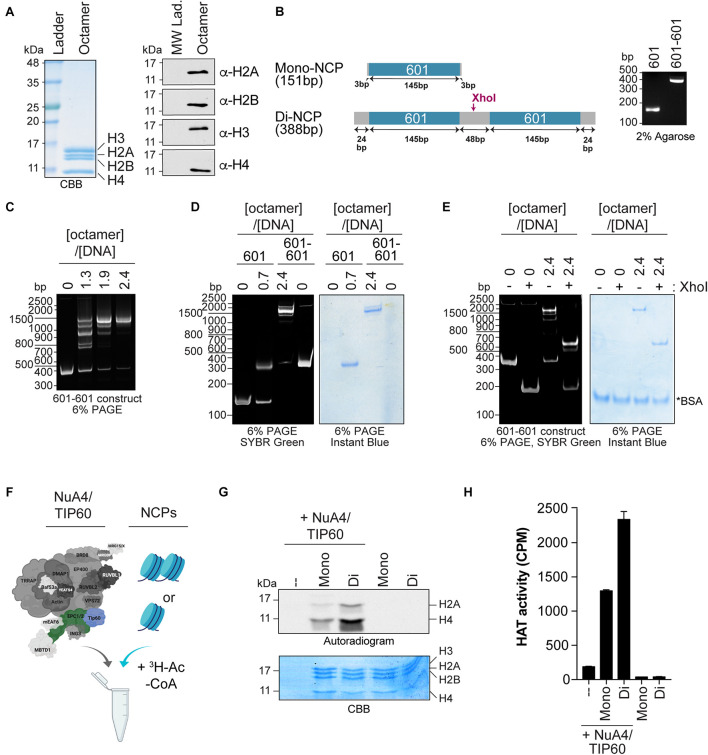
Quantifying the HAT activity of native complexes on recombinant nucleosomes **(A,B)**. Analysis of the octamers and 601 DNA used to reconstitute NCPs. **(A)** Coomassie Brilliant Blue (CBB) staining (left panel) and western blot analyses (right panel) of refolded octamers. **(B)** Schematic representation of the Widom 601 DNA sequence used to wrap mono- and di-NCPs (left panel). The position of the XhoI cleavage site used to validate di-NCP assembly is indicated. Purified DNA obtained from a large prep purification was resolved on a 2% agarose gel and stained with ethidium bromide (right panel). **(C–E)** Analysis of 200 ng of NCP reconstitution on native gels stained with either SYBR green or InstantBlue Coomassie Stain. 200 ng of DNA alone (0) is used as a control. **(C)** Refolding of di-NCPs using the indicated octamer:DNA ratios. **(D)** Mono- and di-NCPs refolded at their optimal octamer:DNA ratios. **(E)** Di-NCPs treated with XhoI for 1 h. The cleaved form of the 601-601 DNA is observed at approximately 200 bp. *BSA used in the reaction. **(F–H)** HAT assay using NuA4/TIP60 complex purified using EPC1-tagged subunit and reconstituted NCPs. The TIP60 subunit, which encompasses the acetyltransferase activity of the complex, is colored in blue and the tagged subunit EPC1 is colored in green. Created with BioRender.com. **(F)** Schematic representation of the HAT assay. **(G)** Autoradiogram showing the HAT activity of 1 μL of purified native NuA4/TIP60 complex on histone H2A and H4. The gel was kept with the X-ray film at –80°C for 4 days. Coomassie Brilliant Blue (CBB) staining was used to show equal loading. **(H)** Quantification of a representative experiment (*n* = 3). HAT reactions were spotted on P81 filters and analyzed with a scintillation counter. Error bars indicate the range between technical duplicate samples.

1. ***Reconstitution of mono- and dinucleosome-containing rNCPs***

Histone purification (A), refolding of core histones into octamers (B), large-scale purification of 601 and 601-601 DNA (C), and nucleosome reconstitution were performed as previously described (Dyer et al., 2003).

A. Histone purification

i. On day 1, transform bacterial expression vectors for histones (human H2A and H2B in pET15b, *X. laevis* H3_C110A_ in pET3d and *X. laevis* H4 in pET3a) into BL21 (DE3) competent cells and plate on lysogeny broth (LB) plates with ampicillin.
ii. On day 2, resuspend colonies and inoculate 1 L of LB plus 100 μg/mL of ampicilin. When cells reach an optical density at 600 nm of 0.5–0.8, induce with 0.4 mM isopropyl β-D-thiogalactoside (IPTG) at 37°C. *An aliquot can be taken prior to addition of IPTG as a negative control for histone induction.*
iii. Pellet the induced bacteria via centrifugation (6,000 × *g*, 15 min, 4°C) 3 h (H2A, H2B, and H3_C110A_) or 1.5 h (H4) post-induction, transfer the bacteria to a 50 mL centrifuge tube in 35 mL of histone wash buffer (50 mM Tris pH 7.5, 100 mM NaCl, and 1 mM EDTA, with 1 mM benzamidine and 5 mM β-mercaptoethanol added fresh), and snap freeze in liquid nitrogen. *An aliquot can be taken prior to centrifugation to monitor histone induction.*
iv. To prepare inclusion bodies, thaw the bacteria in warm water and perform two rounds of freeze-thaw lysis. Add 1 mg/mL lysozyme, nutate 30 min at 4°C, and sonicate until the lysate loses its viscosity. Add histone wash buffer to a total volume of 100 mL, centrifuge (12,000 × *g*, 20 min, 4°C), resuspend the pellet in 75 mL histone wash buffer + 1% Triton X-100, centrifuge (12,000 × *g*, 20 min, 4°C), resuspend the pellet in 75 mL histone wash buffer, and centrifuge again (12,000 × *g*, 20 min, 4°C). Drain the pellet well for the next step. *Cell lysates and purified proteins should be kept on ice at all times unless stated otherwise.*
v. To unfold inclusion bodies, transfer the pellet to a centrifugation tube and dissolve in 260 μL dimethyl sulfoxide for 30 min at room temperature. Mince with a spatula twice during this time. Add 8 mL unfolding buffer (6 M guanidinium HCl and 20 mM Tris pH 7.5, with 5 mM DTT added fresh) and nutate for 1 h at room temperature. Centrifuge (23,000 × *g*, 10 min, room temperature) and retain the supernatant. Rinse the pellet with 1 mL unfolding buffer and centrifuge again (23,000 × *g*, 10 min, room temperature). Pool the supernatants and dialyze them in urea dialysis buffer (7 M urea, 10 mM Tris pH 8, 1 mM EDTA, and 100 mM NaCl, with 5 mM β-mercaptoethanol added fresh, 2× 1 L for 3–4 h and 1× in 1 L overnight^∗^) using dialysis tubing with a cutoff of 3.5 kDa.
vi. To purify the histones, cation exchange chromatography is used. Rinse a clean HiTrap SP HP Sepharose column with 10 mL of water and equilibrate with 20 mL 0.22-μm filtered Buffer A (7 M urea and 20 mM Tris pH 8, with 5 mM β-mercaptoethanol added fresh) at 2 mL/min using a peristaltic pump. Load the dialyzed protein sample and wash with 30 mL Buffer A at 1.5 mL/min. Connect the SP column to an NGC Quest 10 Plus Chromatography System, ensuring that no air bubbles enter the system, and run a linear gradient over 25 CV of 0–100% 0.22-μm filtered Buffer B with a flow rate of 1.5 mL/min and a back-pressure limit of 0.28 MPa. Collect 1.5 mL fractions and monitor the absorbance at 280 nm (A_280_) and conductivity (mS/cm) during elution. Proteins will elute according to their charges, with histones usually eluting at approximately 36 mL and 10 mS/cm.
vii. Resolve eluted fractions from the peaks by 15% SDS-PAGE and pool fractions containing purified histones (hH2A: 14.09 kDa, hH2B: 13.97 kDa, xH3: 15.4 kDa, xH4: 11.37 kDa). Dialyze in 2 mM β-mercaptoethanol in water (3× 4 L for 3–4 h and 1× in 4 L overnight) using dialysis tubing with a cutoff of 3.5 kDa.
viii. Centrifuge any precipitate, use A_280_ values to determine histone concentrations using extinction coefficients (hH2A: 4,470 M^–1^ cm^–1^, hH2B: 7,450 M^–1^ cm^–1^, xH3: 4,470 M^–1^ cm^–1^, xH4: 5,960 M^–1^ cm^–1^) and lyophilize 5 mg of dry histone per 15 mL centrifuge tube using a lyophilizer. Store lyophilized histones at −80°C.
*^∗^ To reduce protein carbamylation by cyanate present in old urea, do not leave your protein in the urea buffer for more than 24 h and deionize the 7 M urea solution for 1 h using MB AG 501-X8 (D) resin prior to adding Tris, EDTA, and NaCl.*

B. Refolding of core histones into octamers

i. Unfold lyophilized histones by nutating them for 30 min at room temperature in 0.22-μm filtered unfolding buffer (20 mM Tris pH 7.5, 7 M guanidine-HCL, and 10 mM DTT added fresh) to a final concentration of ~2 mg/mL.
ii. Combine histones in equimolar ratios in unfolding buffer to a final concentration of ~1 mg/mL and dialyze in 650 mL refolding buffer (10 mM Tris pH 7.5, 2 M NaCl, 1 mM EDTA, and 5 mM β-mercaptoethanol added fresh) in dialysis tubing with a cutoff of 3.5 kDa, 2× 4 h and 1× overnight.
iii. Collect octamers in 15 mL centrifuge tubes, centrifuge to remove any precipitate (4,000 × *g*, 10 min, 4°C), and concentrate samples to <1 mL using Amicon Ultra-0.5 centrifugal filter units with a cutoff of 30 kDa (4,000 × *g*, 10 min, 4°C).
iv. To purify refolded octamers, load the sample on a S200 Superdex 16/60 FPLC column pre-equilibrated with 1.25 CV of 0.22-μm filtered refolding buffer using a 1 mL sample loop. Elute protein complexes at a flow rate of 1 mL/min and a back-pressure limit of 0.5 MPa, and collect 2.5 mL fractions. Monitor the A_280_ during elution. Octamers eluate first, at approximately 62.5 mL, then H3-H4 tetramers and H2A-H2B dimers at approximately 70 and 82 mL, respectively.
v. Resolve eluted fractions from the peaks by 15% SDS-PAGE, pool fractions containing reconstituted octamers (the four histones should be present in equimolar ratios; Figure 2A), and dialyze in 2 mM β-mercaptoethanol in water using dialysis tubing with a cutoff of 3.5 kDa, 3× 4 L for 3–4 h and 1× in 4 L overnight.
vi Concentrate to ≤15 mg/mL with an Amicon Ultra-0.5 centrifugal filter with a cutoff of 30 kDa (4,000 × *g*, 10 min, 4°C), use the A_280_ to determine the concentration using an extinction coefficient (octamer: 44,700 M^–1^ cm^–1^), and store at 4°C for 2–3 months or at −20°C in 50% v/v glycerol for years. Octamers stored in glycerol need to be dialyzed against fresh refolding buffer, and their concentration should be re-evaluated prior to use in NCP reconstitution.
*^∗^ Histones are difficult to quantify accurately. To attain a better idea of the yield, resolve them by SDS-PAGE and stain with Coomassie Brilliant Blue to see if the unfolded histones look equal. Unpaired histones will precipitate during dialysis and reduce the final yield.*

C. Large scale purification of 601 and 601-601 DNA (Figure 2B)

The 601-DNA (Widom DNA) (Lowary and Widom, 1998) and 601-601 DNA are obtained by digesting a 32×601 DNA plasmid (pGEM-3z/601) or E23-L48-E23 plasmid (Kato et al., 2017), which contain a repeated 147-bp nucleosome positioning sequence (Dyer et al., 2003) with EcoRV.

i. Grow DH10β cells transformed with a 601 or 601-601 DNA plasmid in 3× 800 mL of LB plus 100 μg/mL of ampicillin at 37°C and purify the plasmids using a Qiagen GigaPrep kit according to the manufacturer’s instructions.
ii. Digest 3 mg of purified DNA at 37°C with 1,500 units of EcoRV in 15 mL of 1× NEB3 buffer [100 mM NaCl, 50 mM Tris-HCl pH 7.9, 10 mM MgCl_2,_ and 100 μg/mL bovine serum albumin (BSA)] for 16 h to release the positioning sequence DNA. Verify complete digestion by resolving samples on a 1.5% agarose gel.
iii. Transfer the reaction in a 50 mL conical tube. Precipitate backbone DNA (2.5 kbp) by adding 5.1 mL of 40% PEG6000, 2.5 mL of 5M NaCl and 0.15 mL of ultrapure water to reach final concentrations of 9% PEG6000 and 500 mM NaCl in a final volume of 22.50 mL. Incubate 4 h on ice, centrifuge (15,000 × *g*, 30 min, 4°C), carefully decant the supernatant, which contains the smaller DNA fragments (151 bp), and repeat the PEG precipitation for an additional 2 h. Collect the supernatant after centrifugation (15,000 × *g*, 30 min, 4°C). Verify the purity of the DNA fragments by resolving them on a 2% agarose gel.
iv. For 11 mL of supernatant, precipitate the small DNA fragments by adding 27.5 mL of ice-cold 100% EtOH and 0.5 mL of 5M NaCl to reach final concentrations of 70% EtOH and 200 mM NaCl in a final volume of 39 mL. Incubate overnight at 4°C, centrifuge (15,000 × *g*, 30 min, 4°C), transfer the pellet to a 1.5 mL Eppendorf tube in 1 mL of ice-cold 70% EtOH, centrifuge (15,000 × *g*, 5 min, 4°C), remove the supernatant, dry the pellet for 15 min, and resuspend the pellet in 0.2 mL TE buffer (10 mM Tris pH 8.0, 1 mM EDTA). This method usually yields approximately 1 mg of purified nucleosome positioning sequence DNA (Figure 2B).

D. Nucleosome reconstitution

i. Define the molar ratios required for reconstitution to minimize the presence of free DNA. Determine empirically the optimal molar ratios for mono- and dinucleosomes (here, a ratio of 0.7 and 2.4 octamers per DNA were used, respectively) (Figures 2C,D).
ii. Combine 50 μg of octamers (117,760 g/mol) with either 60 μg of 601 DNA (151 bp, 99,660 g/mol) or 45 μg of 601-601 DNA (386 bp, 254,760 g/mol). Final reagent concentrations should be 2 M KCl, ~ 0.7 mg/mL DNA, 10 mM Tris pH 7.5, 1 mM EDTA, and 1 mM DTT. Incubate for 30 min at 4°C, transfer to a 0.5 mL Slide-a-Lyzer with a cutoff of 10 kDa and dialyze against 2 L high salt reconstitution buffer (2 M KCl, 10 mM Tris pH 7.5, and 1 mM EDTA, with 1 mM DTT added fresh) with a decreasing salt gradient over 18 h at 4°C. The gradient is created by constantly pumping low salt reconstitution buffer (0.2 M KCl, 10 mM Tris pH 7.5, and 1 mM EDTA, with 1 mM DTT added fresh) into the 2 L beaker as described (Dyer et al., 2003). Transfer the samples to a fresh beaker containing 400 mL low salt buffer and dialyze for an additional 3 h.
iii. Concentrate to ≤1 mg/mL with an Amicon Ultra-0.5 centrifugal filter with a cutoff of 100 kDa (4,000 × *g*, 10 min, 4°C), determine the concentration using the A_280_, and store at 4°C for 1–2 months. Recombinant nucleosomes can be stored at −80°C in 5–10% v/v glycerol for years.
iv. Resolve 0.2 μg of recombinant nucleosomes on 6% polyacrylamide gels (DNA retardation gels) in 0.2× TBE (18 mM Tris, 18 mM boric acid, and 0.4 mM EDTA, pH 8.0) according to the manufacturer’s instructions (Figure 2D). Samples are prepared in 1× nucleosome loading buffer (8×: 40% sucrose, 0.1% bromophenol blue) and 0.2× TBE. DNA fragments and proteins are visualized by incubating the gel in 1× SYBR green in PBS and in InstantBlue^TM^ Coomassie Stain, respectively, according to the manufacturers’ instructions. Free 601 DNA, reconstituted mono-NCPs, 601-601 DNA, and reconstituted di-NCPs migrate at 151, 350, 350, and 1,500 bp, respectively.
v. Digest 0.2 μg of recombinant nucleosome with 5 units of XhoI (Figure 2E) in 10 μL of 1× CutSmart buffer (50 mM potassium acetate, 20 mM Tris-acetate pH 7.9, 10 mM MgCl_2_-acetate, and 100 μg/mL BSA) at 37°C for 1 h. Resolve on 6% polyacrylamide as above. Cleaved di-NCPs migrate at 650 bp.

***2. HAT assays***

The activity of purified NuA4/TIP60 complex varies between preps. Thus, the amount of NuA4/TIP60 used in HAT assays need to be determined for each preps while doing liquid assays to obtain counts that are in the linear range of the scintillation counter.

E. Reactions (Figure 2F)

i. Perform HAT reactions in a final volume of 15 μL in a 1.5 mL Eppendorf tube. First, combine 0.5 μg of reconstituted NCPs with 1 μL of purified NuA4/TIP60 complex in HAT buffer (50 mM Tris-HCl pH 8, 10 mM sodium butyrate, 5% glycerol, and 0.1 mM EDTA, with 1 mM DTT added fresh). Calculate the KCl molarity in the reaction based on the amounts in the NCP and NuA4/TIP60 complex buffers, and add to a final concentration of 50 mM if necessary. At this point, the reaction volume will be 13.75 μL.
ii. Incubate on ice 10 min.
iii. Add 1.25 μL (0.125 μCi) of H^3^-labeled acetyl-CoA (2.1 Ci/mmol) and incubate at 30°C for 30 min in a water bath.
iv. At this point, the reaction can either be stopped to detect acetylated histones by SDS-PAGE (step 2B) or used in liquid HAT assays to quantify total HAT activity (step 2C).

F. Detection of acetylated histones (Figure 2G)

i. To perform SDS-PAGE, quench the HAT reaction with 5 μL of 4× Laemmli sample buffer and denature the sample for 5 min at 95°C.
ii. Load samples on 15 or 18% SDS-PAGE and migrate for 75–200 min at 160 V, depending on the desired resolution.
iii. Stain the gel with Coomassie Brilliant Blue for 30 min, destain four times for 20 min in 30% methanol and 10% acetic acid, and take an image of the gel.
iv. Destain overnight, incubate the gel for 60 min in EN^3^HANCE, quickly rinse twice in 10% glycerol in water, and incubate for 30 min at room temperature. Dry the gel for 2 h 30 min at 60°C in a gel dryer, place it in an autoradiography cassette with an X-ray film, and store it at −80°C for 1 d to several weeks before developing.

G. Liquid HAT assays (Figure 2H)

i. Microcentrifuge the reactions, then pipette them onto individual P81 phosphocellulose filter papers and air-dry for 30 min.
ii. Wash away free H^3^-labeled acetyl-CoA with 50 mM carbonate buffer pH 9.2 (3.3 mM Na_2_CO_3_ and 47.7 mM NaHCO_3_) three times for 5 min each, then perform an extra quick rinse with acetone. Air-dry for at least 10 min.
iii. Place each P81 paper into a scintillation vial and add 5 mL of EcoLite (+) Liquid Scintillation Cocktail. Measure the H^3^ counts [in counts per minute (CPM)] for 30 min with a scintillation counter.

## Establishment of a Mammalian (m)Anchor-Away System (Figure 3)

**FIGURE 3:**
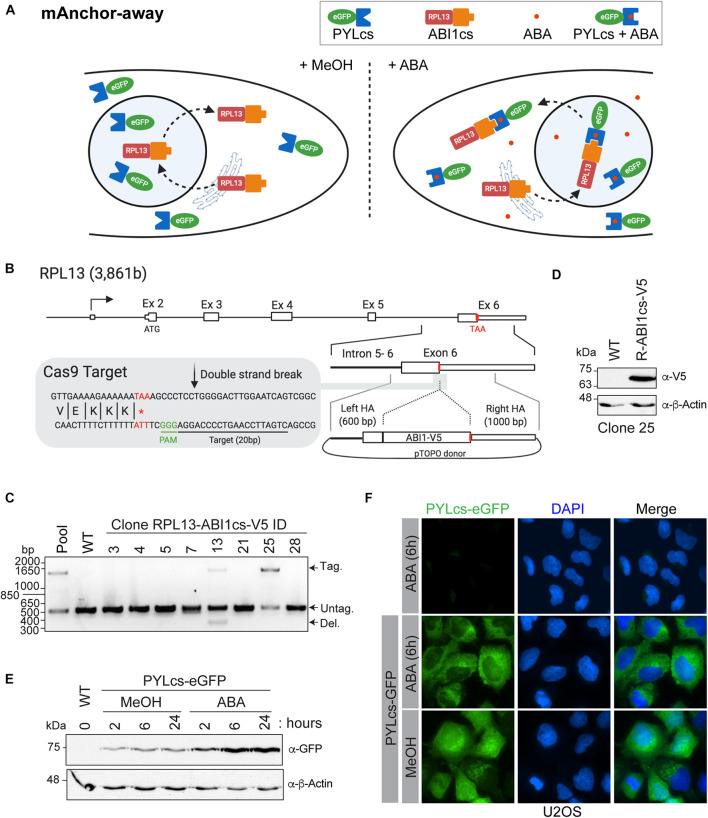
A mammalian anchor-away system to quickly and efficiently deplete chromatin-modifying enzymes from the nucleus **(A).** Schematic of ABA-induced translocation of a nuclear protein to the cytoplasm. In the absence (or presence) of ABA, the ABI1cs-RPL13 fusion protein constantly shuttles between the cytoplasm and the nucleus like most ribosomal proteins, transiting to the nucleolus to assemble ribosome particles with the ribosomal RNA and then going back to the cytoplasm. Upon the addition of ABA, the dimerization of PYLcs-tagged protein with the ABIcs-RPL13 fusion protein triggers its rapid depletion from the nucleus to the cytoplasm, as “anchored away” by the RPL13 ribosomal protein. **(B)** Strategy to establish U2OS cell lines expressing RPL13-ABI1cs-V5 from the endogenous locus. The *RPL13* locus, the Cas9 cleavage site, and the donor construct are indicated. The sequence of the sgRNA targeting *RPL13* is underlined, and the stop codon is indicated in red. HA: homology arm. **(C,D)** Characterization of RPL13-ABI1cs-V5 isogenic cell lines. **(C)** Results of an out-out PCR-base assay conducted on U2OS genomic DNA to detect ABI1cs-V5 integration at the *RPL13* locus. The primers are located in the homology arms and yield a longer PCR product if ABI1cs-V5 is integrated (1,452 bp vs 510 bp for the wild-type allele). In panel **(D)**, whole-cell extracts of wild type U2OS cells and those expressing RPL13-ABI1cs-V5 (clone #25) were western blotted with an anti-V5 antibody to confirm RPL13-ABI1cs-V5 expression (top panel). β-actin was used as a loading control (bottom panel). **(E)** Whole-cell extracts of U2OS RPL13-ABI1cs-V5 cells stably expressing PYLcs-eGFP from the *AAVS1* locus. Samples were collected at the indicated times following treatment with 100 μM ABA (0.1% MeOH was used as a negative control). An anti-GFP antibody was used to detect PYLcs-eGFP (top panel) and β-actin was used as a loading control (bottom panel). **(F)** Immunofluorescence of U2OS cells expressing either RPL13-ABI1cs-V5 alone (clone #25, as a negative control) or RPL13-ABI1cs-V5 and PYLcs-eGFP. Cells were treated with either 100 μM ABA or 0.1% MeOH. DNA was stained with DAPI.

Functional studies of chromatin modifiers using knockdown/knockout approaches are well known to be often associated with undesired secondary effects on important biological processes, such as gene-specific transcription and the cell cycle progression (reviewed in Lalonde et al., 2014). To bypass this issue, we have developed a cellular system to rapidly remove a targeted protein from its usual cellular compartment based on chemically induced proximity. This system was first described as the anchor-away system in yeast (Haruki et al., 2008); however, the use of rapamycin in the system has limited its application in mammalian cells because of its toxicity, instability, and slow clearance. To adapt this method, we took advantage of the S-(+)-abscisic acid (ABA) plant stress pathway, in which the phytohormone ABA binds to pyrabactin resistance (PYR)/PYR1-like (PYL)/regulatory component of ABA receptor family members (Figure 3A). This allows us to use ABA to induce proximity between the interacting complementary surface of PYL (PYLcs), fused to a protein of interest, and proteins fused to the complementary surface of ABA insensitive 1 (ABI1; ABI1cs) (Liang et al., 2011). We chose to fuse the ribosomal protein L13 (RPL13) to ABI1cs to take advantage of its shuttling from the nucleus to the cytoplasm like in the yeast anchor-away system. This would specifically induce the removal from the nucleus of a PYLcs-tagged protein upon ABA addition. CRISPR-Cas9 technology was used to endogenously tag the RPL13 C-terminus with ABI1cs in U2OS cells. Next, a transgene expressing PYL1cs fused to enhanced green fluorescent protein (eGFP) was integrated into the *AAVS1* safe harbor locus (Hockemeyer et al., 2009; DeKelver et al., 2010; Lombardo et al., 2011), and depletion of PYLcs-eGFP from the nucleus was validated by immunofluorescence upon ABA treatment.

1. ***Endogenous tagging of the RPL13 C-terminus using CRISPR/Cas9*** (as described in Dalvai et al., 2015)

A. Generation of single guide (sg)RNA and donor plasmid targeting *RPL13* (Figure 3B)

i Endogenous tagging was performed essentially as described (Doyon and Côté, 2016). Scan your sequence using a web-based CRISPR design tool^1^ to identify sgRNAs that cleave no more than 30 bp away from the stop codon. For *RPL13*, the target site was 5′-CTGATTCCAAGTCCCCAGGA-3′ (Figure 3B). Generate an sgRNA containing an extra G at its 5′ end to accommodate the transcription initiation requirements of the human U6 promoter. A BbsI restriction enzyme site is also added on each end to enable cloning into the pSpCas9(BB)-2A-Puro (pX459) V2.0 vector. Order non-phosphorylated oligonucleotides. Clone the annealed sgRNA into BbsI-digested pX459 V2.0 vector.
ii For the *RPL13* donor plasmid, amplify the homology arms (left: 600 bp and right: 1,000 bp) by polymerase chain reaction (PCR) using genomic DNA isolated from K562 cells. Be sure to mutate the PAM sequence of the sgRNA target sites if it occurs within the homology arms. Assemble using the Zero Blunt TOPO cloning kit. Introduce the ABI1cs sequence (SV-ABAactDA plasmid) (Liang et al., 2011) with a C-terminal V5 tag into the pTOPO-RPL13 donor plasmid via PCR extension overlap using a Gibson Assembly Cloning Kit.

B. Generation of isogenic U2OS cell lines expressing endogenous RPL13-ABI1cs-V5 (Figures 3C,D).

i. Maintain U2OS cells in McCoy’s modified medium supplemented with 10% fetal bovine serum and penicillin-streptomycin at 37°C under 5% CO_2_.
ii. Electroporate 1 × 10^6^ U2OS cells with 2 μg of the donor plasmid, 1 μg of the pX459 V2.0 plasmid expressing eSpCas9, and the gRNA using an Amaxa 4D-Nucleofector and an SE XL Kit, according to the manufacturer’s recommendations.
iii. Expand and select clones by limiting dilution starting 3-d post-transfection in 96-well plates.
iv. Extract genomic DNA with QuickExtract DNA extraction solution and amplify by PCR using the primers 5′-ACTTATGGCAGCGAACCTGA -3′ and 5′-ACCTCCCCACAAGAAAACCG -3′. Resolve on a 1% agarose gel in TAE buffer (40 mM Tris, 1 mM EDTA, and 20 mM glacial acetic acid) to identify the ABI1cs-V5 insertion (Figure 3C). Sequence both alleles to validate accurate gene modification and confirm the absence of indels in the non-targeted allele.
v. Confirm expression of RPL13-ABI1cs-V5 in selected clones by western blotting with an anti-V5 antibody (Figure 3D).

2. ***Generation of U2OS RPL13-ABI1cs-V5 cells expressing PYLcs-eGFP from the AAVS1 safe harbor locus***

i. Amplify PYLcs (SV-ABAactDA plasmid) (Liang et al., 2011) by PCR and clone it in the AAVS1 Puro PGK1 3 × FLAG Twin Strep plasmid using Gibson Assembly Cloning Kit. Amplify eGFP by PCR and replace the 3 × FLAG Twin Strep tag with it via Gibson cloning.

i. To introduce PYLcs-eGFP at the *AAVS1* safe harbor locus via nuclease-driven targeted integration (Dalvai et al., 2015), electroporate 1 × 10^6^ U2OS RPL13-ABI1cs-V5 cells with 1 μg of zinc finger nuclease expression vector (Hockemeyer et al., 2009) and 4 μg of the AAVS1 PYLcs-eGFP donor construct.
ii. Subject cells to puromycin selection for 7 d, starting 3 d post-transfection.
iii. Confirm PYLcs-eGFP expression in the enriched pool following a time-course with 100 μM ABA by western blotting with an anti-GFP antibody (Figure 3E).

3. ***Imaging of ABA-treated RPL13-ABI1cs-V5 U2OS cells***

i. Seed enriched pools of RPL13-ABI1cs U2OS cells with *AAVS1*-integrated PYLcs-eGFP in 6-well plates containing autoclaved coverslips.
ii. At 60% confluency, treat cells with 100 μM ABA (Liang et al., 2011) dissolved in 0.1% methanol (or 0.1% methanol as a control) for 2–24 h.
iii. Wash cells with PBS and fix them with 4% methanol-free formaldehyde for 15 min.
iv. Wash four times with PBS and stain the nuclei using 4′,6-diamidino-2-phenylindole (DAPI; 2 μg/mL). Mount the coverslips on microscope slides using Fluoromount G mounting medium.
v. Capture images with a Nikon Ti Eclipse inverted fluorescence microscope equipped with a Hamamatsu Orca ER camera using 40× or 60× objectives (Figure 3F).

## Results and Discussion

### TAP of Endogenously Tagged NuA4/TIP60 Complex

Studies of native chromatin modifying activities require good yields of purified intact complexes to be obtained by TAP. K562 cells are an excellent model line to purify endogenously tagged chromatin remodelers, as they are permissive to genome editing and tolerate the conditions required to isolate clones. Importantly, large volumes can be cultivated as suspension cultures, which is essential to purify the yields of chromatin modifiers required to perform *in vitro* biochemical analyses and potentially even structural studies. Following the establishment of isogenic K562 cell lines expressing an endogenously TAP-tagged component of the NuA4/TIP60 complex, the FLAG and streptavidin portions of the TAP-tag are used to purify the entire complex in a stepwise manner (Figure 1A). In this specific example, the E1A binding protein p400 (EP400) subunit was used. Nuclear extracts were prepared from K562 cells expressing tagged EP400 as well as a line expressing the 3 × FLAG-2 × Strep tag only (mock). Copurifying proteins were resolved by SDS-PAGE and analyzed by silver staining (Figure 1B). The high sensitivity of silver-stained gels enabled the unambiguous identification of complex subunits, which were not observed in the mock sample. Mass spectrometry analysis of the purified complex confirmed the purification of the entire NuA4/TIP60 complex (Figure 1C).

TAP-based approaches have been used to efficiently purify NuA4/TIP60 complexes using an array of tagged subunits (Ikura et al., 2000; Doyon et al., 2004, 2006; Dalvai et al., 2015; Jacquet et al., 2016). In addition to endogenously tagged proteins, tagged chromatin modifier cDNAs integrated into a safe harbor genomic locus like *AAVS1* can also be used for complex purification (Dalvai et al., 2015). The latter approach is a great alternative, as it allows the expression of near-physiological levels of the tagged protein, is straightforward, and offers high flexibility to study proteins that are difficult to tag at their endogenous locus. It can also be highly useful to compare panels of truncations and mutant proteins in an isogenic setting.

### Characterizing Native Chromatin-Modifying Activities *in vitro*

Chromatin-modifying activities such as acetylation and methylation can be recapitulated *in vitro* using substrates such as peptides mimicking histone tails, purified histones (recombinant or native), rNCPs, and oligonucleosomes isolated from NEs. Of these, rNCPs provide a unique tool to study how nucleosome assembly and specific histone marks affect enzyme activity. Recently, chromatin-remodeling/modifying complexes were found to exhibit different specificity toward mononucleosomes and dinucleosomes, highlighting their higher-order structural specificity (Poepsel et al., 2018; Bhardwaj et al., 2020). We thus used rNCPs to assess whether the activity of the native NuA4/TIP60 complex is affected by the structural differences between the two types of rNCPs.

Mono- and dinucleosomes were reconstituted with octamers of core histone proteins assembled from purified human H2A/H2B and *X. laevis* H3/H4 recombinant histones (Figure 2A), and with short DNA fragments containing either one or two 601 nucleosome positioning sequences (Figure 2B) (Lowary and Widom, 1998; Kato et al., 2017). These sequences have a high affinity for histone octamers and direct nucleosome assembly with high efficiency (Lowary and Widom, 1998; Thåström et al., 1999). Mononucleosome assembly was performed as previously described (Dyer et al., 2003). For the assembly of dinucleosomes, we used the sequence designed by Kato et al. (2017), which contains an internucleosomal 48-bp spacer that accommodates the efficient assembly of two nucleosomes on the donor DNA (Engeholm et al., 2009; Machida et al., 2018). Consistent with previous reports, we observed the formation of a predominant slower-migrating species that corresponds to dinucleosomes with an octamer:DNA molar ratio of 2.4. Nucleosome assembly was monitored by native gel electrophoresis to reveal both the DNA and protein content (Figures 2C,D). Smaller migrating species observed at lower octamer:DNA ratios with the 601-601 DNA were previously found to be due to the assembly of one nucleosome on either one of the positioning elements (Engeholm et al., 2009). The presence of two nucleosomes on the 601-601 DNA in the slower-migrating species observed was further validated by cleaving the linker DNA with XhoI (Figures 2B,E; Machida et al., 2018).

The acetyltransferase activity of purified native NuA4/TIP60 was assessed by *in vitro* HAT assays, in which mono- or dinucleosomes were mixed with the purified complex and H^3^-labeled acetyl-CoA (Figure 2F). The reactions were analyzed by both SDS-PAGE and liquid HAT assays. The autoradiogram revealed that both histones H2A and H4 are more efficiently acetylated when recombinant dinucleosomes were used as substrate (Figure 2G). Coomassie Brilliant Blue staining confirmed that similar amounts of histones were used in each reaction. Liquid HAT assays of the same samples corroborated these results (Figure 2H), which are consistent with a recent observation for the chromatin-modifier polycomb repressive complex 2, which is more active on dinucleosomes than on H3 tails or single nucleosomes (Poepsel et al., 2018). The recent development of a method to assemble rNCPs with various DNA fragments and histone variants further highlights the potential of this approach to characterize the activity of native complexes (Changolkar and Pehrson, 2003; Muthurajan et al., 2016; Sekulic and Black, 2016). For example, rNCPs can be reconstituted with different DNA fragments to study the impacts of different linker DNAs between nucleosomes and in the 5′ and 3′ ends of the template DNA (Engeholm et al., 2009). In addition, specific mutations and PTMs on residues can be engineered on canonical or variant histones prior to reconstitution, allowing very precise questions to be tested experimentally.

### Establishment of an Anchor-Away System for Nuclear Depletion in Mammalian Cells

The mammalian anchor-away system allows the rapid and robust depletion of a PYLcs-tagged protein from the nucleus (Figure 3A). As long-term depletion of NuA4/TIP60 complex subunits leads to cell toxicity (Gorrini et al., 2007; Fazzio et al., 2008; Hu et al., 2009; Steunou et al., 2014; Numata et al., 2020), this system provides a great alternative to study its roles in biological processes, including DNA repair (Jacquet et al., 2016). In this example, modifier depletion immediately prior to DNA break induction would be useful to separate an acetyltransferase’s transcriptional functions from its role acetylating histones surrounding the break. To establish this system in mammalian cells, we fused an ABI1cs-V5 tag to the C-terminal domain of the ribosomal protein RPL13 (homologous to the anchor used in the yeast system) using a CRISPR/Cas9-driven approach. A sgRNA targeting the 3′-end of exon 6 was designed to target the nuclease near the stop codon (Figure 3B). A donor molecule containing the TAP tag and homology arms for RPL13 was used to integrate the tag and delete the endogenous stop codon. Following transfection into U2OS cells, tag incorporation was detected in the pool and in two isolated clones by PCR (Figure 3C). Accurate gene modification was also confirmed by western blot analysis (Figure 3D) and by sequencing, as previously described (Dalvai et al., 2015). The PYLcs-eGFP fusion protein was integrated at the *AAVS1* locus in U2OS-RPL13-ABI1cs-V5 clone 25, and its expression was confirmed by western blot analysis following the addition of ABA (or MeOH as a negative control; Figure 3E). Using immunofluorescence, we observed that adding ABA triggered the nuclear exclusion of eGFP 6 h after treatment (Figure 3F). Interestingly, the PYLcs-eGFP fusion was less abundant by western blot before ABA addition, suggesting that the fusion is expressed at low levels and stabilized upon complexing with RPL13-ABIcs-V5 (Figure 3D). Although these results are promising, further experiments will be required to validate that the fusion between PYLc and a ribosomal protein can efficiently deplete subunits of chromatin-modifying enzymes from the nucleus, to establish the kinetics of this process and to determine if the fusion impact the endogenous function of RPL13. The fact that we were unable to isolate a clone with homozygous tagged RPL13 (Figure 3C) raises a concern about the impact of the fusion on the cellular function of the protein. The main advantage of this system compared to other systems based on proteasome degradation (AID/Tir1 and dTAG approaches (Nabet et al., 2018; Nishimura et al., 2020; Yesbolatova et al., 2020) is that the recovery of essential nuclear protein should be faster in the absence of the drug as no degradation is involved. It is thus expected that it will have less impact on cellular fitness during experiments. Nonetheless, our results provide proof of concept of the mammalian anchor-away system’s great potential to enable temporal examination of the specific functions of essential chromatin modifiers.

## Conclusion

The detailed step-by-step protocols provided here will be helpful to researchers interested in rapidly characterizing native chromatin modifying complexes. Of course, the enzymatic assays used will differ depending on the PTM deposited, and NCP composition should be modified depending on the presumed target or hypothesis being tested (e.g., H3.3 variants vs H3.1, H2A.Z/H2A.X vs H2A). Streamlining these approaches within research teams will greatly expand the experimental angles available to address scientific questions about chromatin-based molecular mechanisms.

## Materials and Equipment

### Reagents

E23-L48-E23 plasmid (Kato et al., 2017)
3 × FLAG peptide (Sigma, Cat. No. F4799)
AAVS1 Puro PGK1 3 × FLag Twin Strep Plasmid (Addgene, Cat. No. 68375)
Abscisic acid (Sigma, Cat. No. 5.30339)
Acetic acid (Anachemia, Cat. No. 000598-460)
Acetonitrile (Sigma, Cat. No. 271004)
Agar A (BioBasic, Cat. No. FB0010)
Ampicillin (Bioshop, cat. No. AMP201.100)
Anti-FLAG M2 affinity beads (Sigma, Cat. No. F1804)
Anti-GFP (Roche, Cat. No. 11814460001)
Anti-V5 antibody (Abcam, Cat. No. SV5-Pk1)
Aprotinin (Sigma, Cat. No. A3886)
BbsI (NEB, Cat. No. R0539)
Benzamidine hydrochlorate hydrate (Sigma, Cat. No. B6506)
β-mercaptoethanol (Sigma, Cat. No. M3148)
β-glycerophosphate (Sigma, Cat. No. G9422)
Bolt 4–12% Bis-Tris gels (Thermo Fisher Scientific, Cat. No. NW04120BOX)
Bolt 12% Bis-Tris gels (Thermo Fisher Scientific, Cat. No. NW00122BOX)
Boric acid (Sigma, Cat. No. B6768)
Bromophenol blue (Bioshop, Cat. No. BRO222)
Citric acid (Sigma, Cat. No. 251275)
Coomassie Brilliant Blue (Bioshop, Cat. No. CBB555)
Coverslips (FisherBrand, Cat. No. 12541B)
CutSmart Buffer (NEB, Cat. No. B7204S)
D-Biotin (ThermoFisher, Cat. No. B20656)
DH10ß (Thermofisher, Cat. No. EC0113)
Dimethyl sulfoxide (Sigma, Cat. No. D8418)
DNA retardation gel (Life Technology, Cat. No. EC6365BOX)
DTT (Bioshop, Cat. No. DTT002)
EcoRV (NEB, Cat. No. R3195L)
EDTA (Sigma, Cat. No. E5134)
EN3HANCE solution (PerkinElmer, Cat. No. 6NE9701)
Ethanol, 100% (Commercial Alcohols, Cat. No. P016EAAN)
Fetal bovine serum (ThermoFisher, Cat. No. 12483020)
Filter paper – P81 (Sigma, Cat. No. Z742570)
Fluoromount G (eBioscience, Cat. No. 00-4958-02)
Formaldehyde (Sigma, Cat. No. 252549)
Gibson Assembly kit (NEB, Cat. No. E5510S)
Glutaraldehyde (Sigma, Cat. No. G6403)
Glycerol (Bioshop, Cat. No. GLY001)
Guanidinium-HCl (BioBasic, Cat. No. GB0242)
H^3^-labeled acetyl-CoA (PerkinElmer Life Sciences, cat. No. NET290050UC)
HEPES (Bioshop, Cat. No. HEP001.1)
HEPES, 1 M, for cell culture (Life Technologies, Cat. No. 15630080)
HiTrap SP HP Sepharose columns (GE Healthcare, Cat. No. 45-100-294)
InstantBlue^TM^ Coomassie Stain (Bioshop, Cat. No. CBB555.25)
Isopropyl β-D-thiogalactoside (Sigma, Cat. No. I6758-10G)
K562 cells (ATCC, Cat. No. CCL-243)
KCl, reagent grade (Bioshop, Cat. No. POC308)
Leupeptin (Sigma, Cat. No. 78435)
EcoLite (+) Liquid Scintillation Cocktail (MP Biomedicals, Cat. No. 0188247504)
Lysozyme from chicken egg (Sigma, Cat. No. L6876)
MB AG 501-X8 (D) resin (BioRad, Cat. No. 1436425)
McCoy’s Modified Medium (ThermoFisher, Cat. No. 16600108)
Methanol (Fisher Chemical, Cat. No. A412)
Methanol Free 16% Formaldehyde (ThermoFisher, Cat. No. 28908)
MgCl_2_ ACS reagent grade (Bioshop, Cat. No. MAG510)
Microscope slides (FisherBrand, Cat. No. 22-034-486)
MOPS (Sigma, Cat. No. M3183)
Na_2_CO_3_ (Sigma, Cat. No. 451614)
NaCl (Bioshop, Cat. No. SOD001.5)
NaF (Sigma, Cat. No. 201154)
NaHCO_3_ (Sigma, Cat. No. S6014)
Orthovanadate (Sigma, Cat. No. S6508)
P81 phosphocellulose filter paper (Sigma, Cat. No. Z742570)
pGEM-3z/601 (Addgene, Cat. No. 26656)
PEG6000 (Cederlane, Cat. No. 8.07491.1000)
Penicillin-Streptomycin (ThermoFisher, Cat. No. 15140122)
Pepstatin A (Sigma, Cat. No. P5318)
Pet15b vector (EDM Millipore, Cat. No. 69661)
Pet28a Synthetic Human H2A.1 (Addgene, Cat. No. 42634)
Pet28a Human H2B.1 (Addgene, Cat. No. 42630)
Pet3d Xenopus H3_C110A_ and Pet3a Xenopus H4 plasmids (a kind gift from Cheryl Arrowsmith)
PMSF (Thermo Fisher Scientific, Cat. No. 36978)
pSpCas9(BB)-2A-Puro (pX459) V2.0 vector (Addgene, Cat. No. 62988)
Puromycin (Sigma, Cat. No. P8833)
Qiagen GigaPrep kit (Qiagen, Cat. No. 12191)
QuickExtract DNA extraction solution (Epicentre, Cat. No. QE09050)
RPMI medium (Life Technologies, Cat. No. 21870-092)
Sepharose CL-6B resin (Sigma, Cat. No. CL-6B-200)
SE XL Kit, nucleofection (Lonza, Cat. No. V4LC-2020)
Silver nitrate (Sigma, Cat. No. S8157)
Sodium butyrate (Sigma, Cat. No. B5887)
Sodium carbonate (Sigma, Cat. No. 451614)
Strep-Tactin Superflow Sepharose affinity matrix (IBA, Cat No. 2-1206-010)
Sucrose, biotechnology grade (Bioshop, Cat. No. SUC700)
SV-ABAactDA plasmid (Addgene, Cat. No. 38247)
SYBR green (ThermoFisher, Cat. No. S7563)
Sypro^TM^ Ruby protein gel stain (Bio-Rad, Cat. No. 1703125)
Tris base (Bioshop, Cat. No. TRS001.5)
Triton X-100 (BioBasic, Cat. No. TB0198)
Tryptone powder (BioBasic, Cat. No. G211)
Tween-20 (Bioshop, Cat. No. TWN508)
U2OS cells (ATCC, HTB-96)
Urea, reagent grade (Bioshop, Cat. No. URE002)

Yeast extract (BioBasic, Cat. No. G0961)
Zero Blunt pTOPO cloning kit (Life Technology, Cat. No. 450245)

### Equipment

Amicon Ultra-0.5 centrifugal filter units (Millipore, Cat. No. UFC503024)
Bioruptor (Diagenode)
Dounce homogenizer with a type B pestle (Thomas Scientific, Cat. No. 1229H80)
Dialysis tubing with a cut off of 3.5 kDa (Thermo Scientific, Cat. No. REF68035)
Electrophoresis and blotting apparatus (Biorad, Cat. Nos. 1658001FC and 1703930)
Labconco FreeZone 1 Liter Benchtop Freeze Dry System (Cat. No. 7740020)
LS 6500 Multi-purpose Scintillation Counter (Beckman Coulter)
Nikon Ti Eclipse fluorescence microscope with a Hamamatsu Orca ER camera
NGC Scout 10 Plus and fractionator (BioRad)
Micro Bio-Spin columns (BioRad, Cat. No. 7326204)
Nutator
Optima LE-80K Ultracentrifuge (Beckman-Coulter)
Peristaltic pump (Buchler Instruments Polystaltic Pump)
Poly-Prep chromatography columns (Bio-Rad, Cat. No. 7311550)
S200 HiLoad 16/600 Superdex FPLC columns (GE Healthcare, Cat. No. 28-9893-35)
Slide-A-Lyzer 0.5 mL with a cut off of 10 kDa (Life Technology, Cat. No. 66383)
Scintillation vials (Fisher, Cat. No. 03-337-20)
Sorvall LYNX 4000 Superspeed Centrifuge and tubes (Thermo Scientific)
Spinner Flasks, 3L (Fisher, Cat. No. 10203E)
UV spectrophotometer (Nanodrop One^*c*^, Thermo Scientific)
Water bath

### Solutions

**Table:** 

Buffer A	7 M urea, 20 mM Tris pH 8, add 5 mM β-mercaptoethanol fresh (just before use)
Buffer B	7 M urea, 20 mM Tris pH 8, 1 M NaCl, add 5 mM β-mercaptoethanol fresh
Carbonate developing solution	283 mM sodium carbonate, 0.0185% formaldehyde
CutSmart buffer	50 mM potassium acetate, 20 mM Tris-acetate pH 7.9, 10 mM MgCl_2_-acetate, 100 μg/mL BSA
HAT buffer	50 mM Tris-HCl pH 8, 10 mM sodium butyrate, 5% glycerol, 0.1 mM EDTA, add 1 mM DTT fresh
High salt buffer	10 mM HEPES pH 7.9, 1.5 mM MgCl_2_, 1.2 M KCl, 25% glycerol, 0.2 mM EDTA, add 0.2 mM PMSF and 0.5 mM DTT fresh
High salt reconstitution buffer	2 M KCl, 10 mM Tris pH 7.5, 1 mM EDTA, add 1 mM DTT fresh
Histone wash buffer	50 mM Tris pH 7.5, 100 mM NaCl, 1 mM EDTA, add 1mM benzamidine and 5 mM β-mercaptoethanol fresh
Hypotonic buffer	10 mM HEPES pH 7.9, 1.5 mM MgCl_2_, 10 mM KCl, add 0.2 mM PMSF and 0.5 mM DTT fresh
Low salt buffer	10 mM HEPES pH 7.9, 1.5 mM MgCl_2_, 20 mM KCl, 25% glycerol, 0.2 mM EDTA, add 0.2 mM PMSF and 0.5 M DTT fresh
Low salt reconstitution buffer	0.2 M KCl, 10 mM Tris pH 7.5, 1 mM EDTA, add 1 mM DTT fresh
MOPS SDS running buffer	50 mM MOPS, 50 mM Tris, 0.1% SDS, 1 mM EDTA
NEB3 buffer	100 mM NaCl, 50 mM Tris-HCl pH 7.9, 10 mM MgCl_2_, 100 μg/mL BSA
Nucleosome loading buffer (8×)	40% sucrose, 0.1% bromophenol blue
Refolding buffer	10 mM Tris pH 7.5, 2 M NaCl, 1 mM EDTA, add 5 mM β-mercaptoethanol fresh
Running buffer	25 mM Tris, 50 mM glycine, 0.1% SDS
TAE buffer	40 mM Tris, 1 mM EDTA, 20 mM glacial acetic acid
TAP buffer	20 mM HEPES-KOH pH 7.9, 300 mM KCl, 1.5 mM MgCl_2_, 0.2 mM EDTA, 10% glycerol, add 10 mM sodium butyrate, 10 mM β-glycerophosphate, 1 mM PMSF, 5 mM NaF, 100 μM orthovanadate, 2 μg/mL leupeptin, 2 μg/mL pepstatin, and 5 μg/mL aprotinin fresh
TAP wash buffer #1	20 mM HEPES-KOH pH 7.9, 300 mM KCl, 0.1% Tween-20, 10% glycerol, add 1 mM DTT, 10 mM sodium butyrate, 10 mM β-glycerophosphate, 1 mM PMSF, 5 mM NaF, 100 μM orthovanadate, 2 μg/mL leupeptin, 2 μg/mL pepstatin, and 5 μg/mL aprotinin fresh
TAP wash buffer #2	20 mM HEPES-KOH pH 7.9, 150 mM KCl, 0.1% Tween-20, 10% glycerol, add 1 mM DTT, 10 mM sodium butyrate, 10 mM β-glycerophosphate, 1 mM PMSF, 5 mM NaF, 100 μM orthovanadate, 2 μg/mL leupeptin, 2 μg/mL pepstatin, and 5 μg/mL aprotinin fresh
TBE (0.2×)	18 mM Tris, 18 mM boric acid, 0.4 mM EDTA, pH 8.0
Unfolding buffer	20 mM Tris pH 7.5, 7 M guanidine-HCL, add 10 mM DTT fresh
Urea dialysis buffer	7 M urea, 10 mM Tris pH 8, 1 mM EDTA, 100 mM NaCl, add 5 mM β-mercaptoethanol fresh.

## Data Availability Statement

The original contributions presented in the study are included in the article/supplementary material, further inquiries can be directed to the corresponding author/s.

## Author Contributions

AF-T and JC conceived the original idea and supervised the project. MG, CL, XC, and FD-G performed initial protocol optimization. MG and CL performed the experimental work presented in this manuscript. AF-T and JC wrote the manuscript with contributions from MG and CL. All authors contributed to the article and approved the submitted version.

## Conflict of Interest

The authors declare that the research was conducted in the absence of any commercial or financial relationships that could be construed as a potential conflict of interest.

## Publisher’s Note

All claims expressed in this article are solely those of the authors and do not necessarily represent those of their affiliated organizations, or those of the publisher, the editors and the reviewers. Any product that may be evaluated in this article, or claim that may be made by its manufacturer, is not guaranteed or endorsed by the publisher.

## Footnotes

1
http://crispr.mit.edu/
